# Pregnancy exacerbated lymph node metastasis of well differentiated thyroid carcinoma: A case report

**DOI:** 10.1097/MD.0000000000028264

**Published:** 2021-12-23

**Authors:** I-Ju Tu, Shih-Ming Huang, Ying-Ren Chen

**Affiliations:** a Department of Obstetrics and Gynecology, Taipei Medical University Hospital, Taipei, Taiwan; b Department of Surgery, National Cheng Kung University Hospital, College of Medicine, National Cheng Kung University, Tainan, Taiwan; c Department of surgery, Chang Bing Show Chwan Memorial Hospital, Changhua, Taiwan; d Department of Pathology, National Cheng Kung University Hospital, College of Medicine, National Cheng Kung University, Tainan, Taiwan.

**Keywords:** differentiated thyroid cancer, gene mutation, pregnancy

## Abstract

**Introduction:**

Differentiated thyroid carcinoma is the second most frequently diagnosed cancer during pregnancy, second to breast cancer. Pregnancy can cause an increase in the size of existing thyroid nodules due to the similar structure of placental human chorionic gonadotropin and thyroid stimulating hormone. However, the impact of pregnancy on malignant thyroid tumors is still unclear.

**Patient concerns:**

We report a 27-year-old woman with initial thyroid follicular carcinoma was managed with total thyroidectomy and radioiodine therapy. Tumor recurrences with right neck lymph node enlargement were noted during the first and third trimester of pregnancy two years after initial diagnosis.

**Diagnosis:**

Right neck lymph node dissection was performed for two episodes of recurrence and the pathology revealed both metastatic papillary thyroid carcinoma, follicular variant but with different pathologic features. And next-generation DNA sequencing of 275 cancer-related genes, which was a commercial set, including common mutations in thyroid cancer revealed only point mutations with unknown clinical correlation.

**Intervention:**

For the first recurrence during pregnancy, right neck lymph node dissection was performed at the second trimester of pregnancy. As for the second recurrence in the third trimester of pregnancy, the patient received right neck lymph node dissection with radioiodine therapy one month after uncomplicated delivery.

**Outcomes:**

After complete treatment with surgery and radioiodine therapy, the serum thyroglobulin level was 10 ng/ml. During two-year regular follow-ups with serum thyroglobulin and ultrasound, no more recurrence was noted.

**Conclusion:**

Pregnancy in differentiated thyroid cancer survivors should be managed and monitored with caution, especially when cancer recurrence is noticed. Further studies are recommended to investigate these previously unreported gene mutations associated with thyroid cancer.

## 1 Introduction

Thyroid cancer is the second most common cancer diagnosed during pregnancy and in the postpartum period after breast cancer.^[1,2]^ A previous study showed that pregnancy is associated with an increase in the size of preexisting thyroid nodules as well as new thyroid nodule formation, which may predispose to multinodular goiter in later life.^[3]^ The 2017 American Thyroid Association Guideline states that pregnancy does not pose a risk for differentiated thyroid cancer recurrence in women without structural or biochemical disease present prior to the pregnancy but may stimulate thyroid cancer growth in patients with known structural or biochemical disease present at the time of conception.^[4]^ Hence, monitoring is required, such as ultrasound and measurement of serum thyroglobulin levels. Here we report a case of well differentiated thyroid carcinoma with exacerbated lymph node metastasis during the first and third trimester of pregnancy.

## 2 Case report

**Figure 1:**
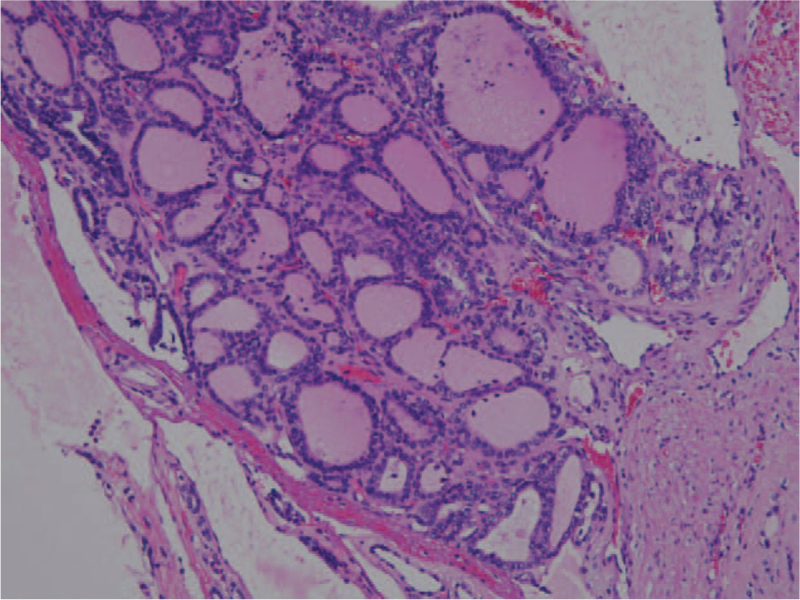
The initial thyroidectomy was diagnosed of follicular carcinoma, with follicular cells in solid nests and mostly micro-follicular patterns.

**Figure 2:**
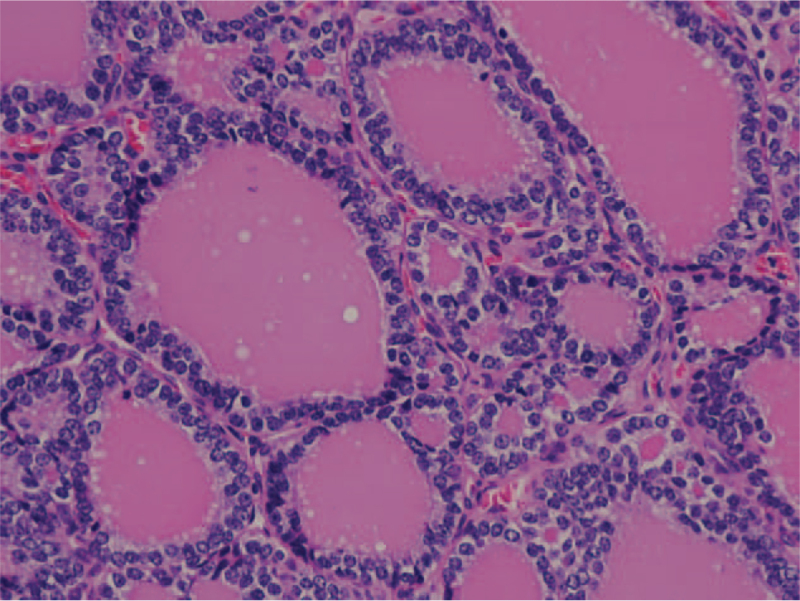
The recurrent metastatic cervical lymph node was diagnosed of papillary thyroid carcinoma, follicular variant (PTC-FV). Pathologic section showed oval nuclei with finely dispersed chromatins, arranged in diffuse follicular pattern.

**Figure 3:**
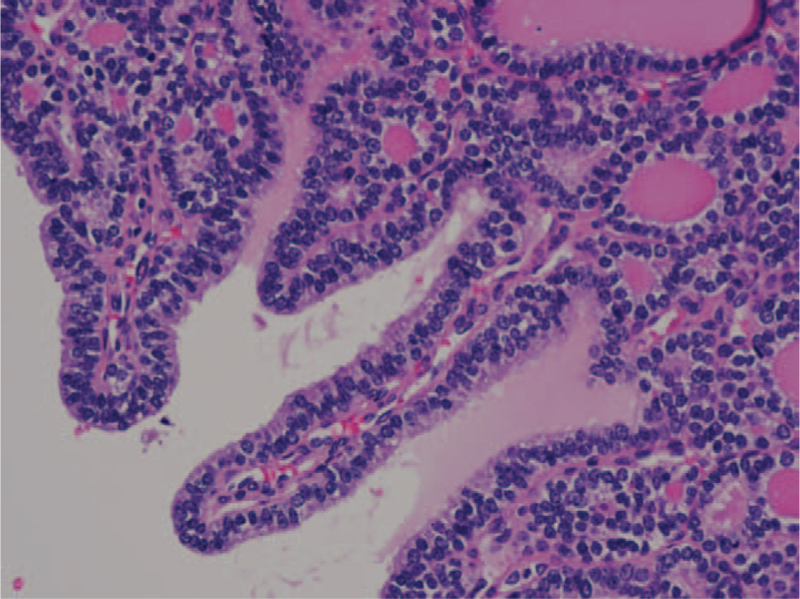
The recurrent metastatic cervical lymph node was diagnosed of papillary thyroid carcinoma, follicular variant (PTC-FV). Pathologic section showed few papillae formation.

**Figure 4:**
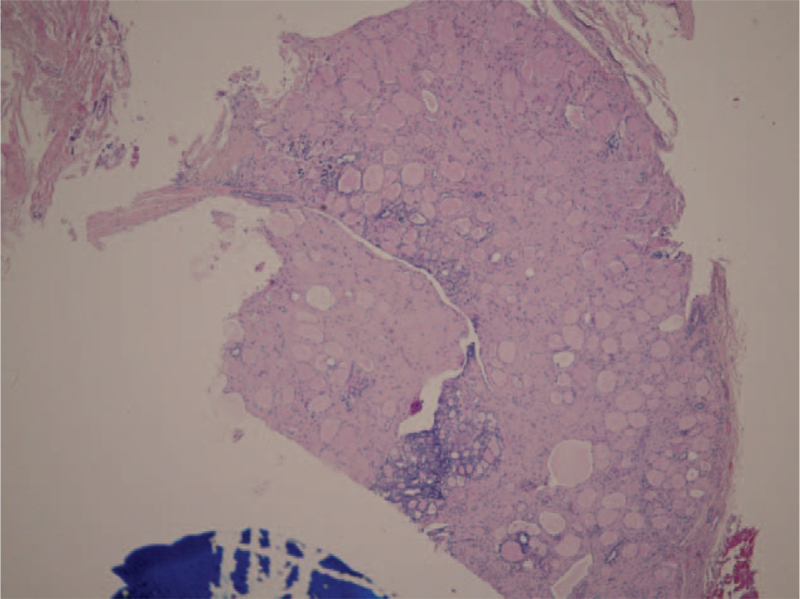
The second recurrent lymph node showed that architecture was totally replaced by tumor cells arranged in mixed macro- and micro-follicular patterns without obvious papillary structure.

A 27-year-old Taiwanese woman (gravida 0, para 0, abortus 0) was diagnosed with thyroid follicular carcinoma. (Fig. 1) The initial presentation was a right palpable mass around 10 cm in diameter as measured by ultrasound. After total thyroidectomy and 100 mCi-131 treatments, her serum thyroglobulin was reduced to ≤2 ng/ml and no neck lymph node enlargement was noted during the two-year follow-up. Two years later, at the age of 29, she presented with two right neck lymph node enlargements with a maximal diameter of 1.4 cm. Serum thyroglobulin had increased to 5.5 ng/ml and ultrasound-guided FNA aspiration needle washout for thyroglobulin showed >500 ng/ml. A scheduled operation was postponed due to an unplanned pregnancy at 8th gestational age. Regular follow up with ultrasound showed an increased number and size of the lymph nodes with a maximal diameter up to 6 cm, and serum thyroglobulin increased to 123 ng/ml within 2.5 months. At the gestational age of 18 weeks, a right neck lymph node dissection was performed, revealing metastatic papillary thyroid carcinoma, follicular variant (Figs. 2 and 3) and serum thyroglobulin was 20 to 40 ng/ml post-operation. However, lymph node enlargement over the right thyroid bed was noted by ultrasound, and serum thyroglobulin was 55 ng/ml at the 39th gestation week. One month after an uncomplicated full-term delivery, she underwent another right neck lymph node dissection, with the pathology showing a mostly follicular structure with very little papillary pattern (Fig. 4). After 150 mCi-131 treatment, serum thyroglobulin was 10 ng/ml. Due to the difficulty of the pathologic diagnosis and aggressive behavior of the tumor, the first recurrent lymph node tissue was sent for next-generation DNA sequencing (NextSeq500, Illumina), revealing four point mutations: DICER1:p. L1642fs, DICER1:p. E1813G, BCL6: p.R575C, and PMS1:p.P889S.

## 3 Discussion

Due to the structural similarity of placental human chorionic gonadotropin (hCG) and thyroid stimulating hormone (TSH), hCG stimulates the TSH receptor causing increased thyroid gland activity during pregnancy.^[1]^ Serum hCG is produced by the syncytiotrophoblast and is undetected in non-pregnant adults. After conception, serum hCG increases from the day of implantation with a doubling time of 1.4 to 2.0 days, reaching a peak at 60 to 70 days and can be up to 100,000 mIU/mL. It then declines slowly, reaching a plateau at around gestational age 16 weeks. Although the serum concentration gradually decreases in the third trimester, it is still much higher than normal and takes 6 to 70 days to return to undetectable levels after delivery.^[5]^

Current guidelines advise delaying surgery until the postpartum period as there is no adequate evidence showing that pregnancy worsens the cancer prognosis. However, surgery is recommended if the disease is aggressive, with aggressive or locally advanced histology, metastatic cervical lymph nodes, severe compressive symptoms, or significant growth of malignant nodules.^[4,6]^ If surgery in pregnancy is indicated, it should be performed during the second trimester to minimize complications, such as altered organogenesis and miscarriage in the first trimester, preterm labor, or delivery in the third trimester.^[4]^ In our case, due to the rapidly growing tumor size and number as well as increased serum thyroglobulin during the first trimester, we operated in her second trimester. The increase and fluctuation of hCG during pregnancy may affect thyroid cancer recurrence, though the impact and relationship are still unclear.

The initial thyroid pathology report showed follicular carcinoma (Fig. 1), however, the first recurrent cervical metastatic lymph node showed a diffuse follicular pattern (Fig. 2) with few papillae formation (Fig. 3). Although there were no classic papillary nuclear features, such as pseudoinclusion, owing to few papillae formation and clinical nodal metastasis, the final pathologic diagnosis of the recurrent metastatic thyroid cancer was papillary thyroid carcinoma. The second lymph node dissection showed that the lymph node architecture was replaced by tumor cells arranged in mixed macro- and micro-follicular patterns without an obvious papillary structure, which resembled nodular goiter of the thyroid (Fig. 4). Similar nuclear features as the previous tumor were identified. Considering the previous high-dose I-131 treatment, no normal thyroid architecture would be left, thus, together with her previous history, metastatic papillary thyroid carcinoma, follicular variant was considered.

Although the common gene mutations associated with thyroid cancer such as KRAS, NRAS, HRAS, BRAF, PIK3CA, RET, and MMR-relating gene were not detected in the next-generation DNA sequencing, four point mutations were identified: DICER1: p. L1642fs, DICER1: p. E1813G, BCL6: p.R575C, and PMS1: p.P889S. Among them, *DICER* and *BCL* genes are associated with the pathogenic phenotype according to the Clinvar genome resources of the National Center for Biotechnology Information. It is possible that these point mutations are related to the aggressive behavior and difficult diagnosis of the unusual pathological features in this case. Future studies are needed to investigate the genetic alterations of thyroid cancer, especially those without common gene mutations but with an aggressive phenotype behavior.

In conclusion, pregnancy stimulates the fluctuation of multiple hormones, including hCG, which have a similar structure to TSH. Therefore, pregnancy in differentiated thyroid cancer survivors should be managed and monitored with caution, especially when cancer recurrence is noticed. Further studies are recommended to investigate these previously unreported gene mutations associated with thyroid cancer.

## Author contributions

**Conceptualization:** I-Ju Tu, Shih-Ming Huang, Ying-Ren Chen.

**Data curation:** I-Ju Tu, Shih-Ming Huang, Ying-Ren Chen.

**Formal analysis:** I-Ju Tu, Shih-Ming Huang, Ying-Ren Chen.

**Investigation:** I-Ju Tu, Shih-Ming Huang, Ying-Ren Chen.

**Methodology:** I-Ju Tu, Shih-Ming Huang, Ying-Ren Chen.

**Project administration:** I-Ju Tu, Shih-Ming Huang.

**Resources:** Shih-Ming Huang.

**Supervision:** Shih-Ming Huang, Ying-Ren Chen.

**Validation:** Shih-Ming Huang.

**Visualization:** Shih-Ming Huang.

**Writing – original draft:** I-Ju Tu, Ying-Ren Chen.

**Writing – review & editing:** I-Ju Tu, Shih-Ming Huang.
